# A pull-down assay using DNA/RNA-conjugated beads with a customized competition strategy: An effective approach to identify DNA/RNA binding proteins

**DOI:** 10.1016/j.mex.2020.100890

**Published:** 2020-04-13

**Authors:** Hongyan Sui, Qian Chen, Tomozumi Imamichi

**Affiliations:** Laboratory of Human Retrovirology and Immunoinformatics, Frederick National Laboratory for Cancer Research, Bldg. 550, Rm 126,1050 Boyles Street, Frederick, MD 21702 United States

**Keywords:** A pull-down assay, DNA/RNA-conjugated beads, Competition, Cytosolic DNA/RNA sensors

## Abstract

Innate immune response is insisted upon detection of foreign intracellular DNA or RNA derived from viruses and bacteria. This reaction is important to initiate an effective protective response for the host cells. This crucial step is induced by cytosolic nucleic acids sensors/binding proteins, which triggers the production of type I or type III interferons (IFNs) and proinflammatory cytokines such as Interleukin 6 (IL-6). The identification of these cytosolic DNA or RNA sensors is a key step in understanding the signaling pathways triggered by those pathogens. Here we describe an effective approach to identify potential known/novel DNA or RNA sensors: a pull-down assay using DNA/RNA-conjugated beads with a customized competition strategy, which conferred a more effective and efficient way to determine the interaction between DNA/RNA and the sensor protein(s), therefore greatly improves the progress to investigate potential novel cytosolic DNA or RNA sensors/ binding proteins

• The customized method makes the traditional pull-down assays more effective and efficient to identify DNA/RNA binding protein(s).
• With the competitor of your choice, the method provides specific information about the competitive binding between DNA/RNA and binding proteins.

## Graphical abstract

**Figure:**
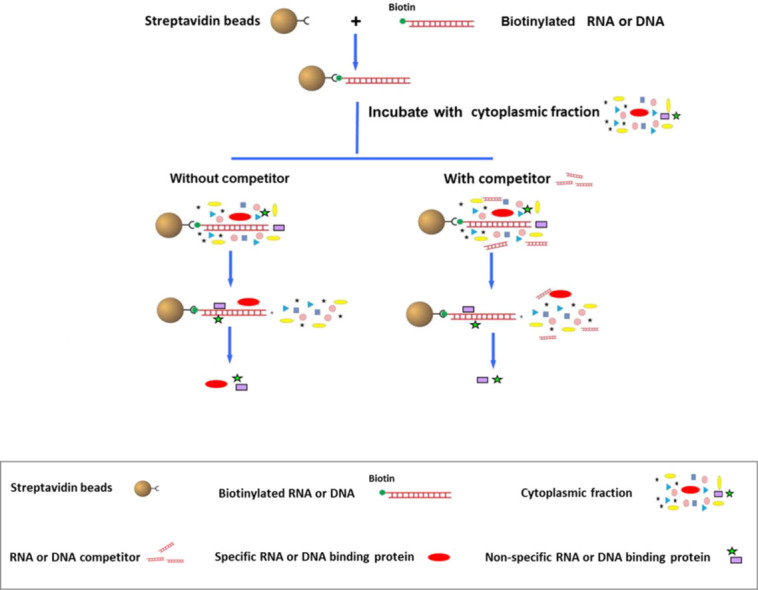

**Table:** Specifications Table

Subject Area	*Select one of the following subject areas:* *•Immunology and Microbiology*
More specific subject area:	*Innate immunology*
Method name:	*A pull-down assay using DNA/RNA-conjugated beads with a customized competition strategy*
Name and reference of original method	*If applicable, include full bibliographic details of the main reference(s) describing the original method from which the new method was derived.*
Resource availability	*If applicable, include links to resources necessary to reproduce the method (e.g. data, software, hardware, reagent)*

*Method details:

• Materials
  1. Biotinylated-RNA or DNA (IDT, Coralville, IA, USA)
  2. Unconjugated-RNA or DNA (IDT, Coralville, IA, USA)
  3. Nuclear Extract kit (Active Motif, Carlsbad, CA, USA)
  4. Dynabeads™ M-280 Streptavidin (Invitrogen, Oslo, Norway)
  5. DynaMag™ Magnet (Invitrogen, Oslo, Norway)
  6. Mixing device with tilting and rotation
• Buffers and solutions
  1. Binding and washing (B&W) buffer (2X): 10 mM Tris–HCl (pH 7.5), 1 mM EDTA, 2 M NaCl;
  2. For RNA applications: Solution A (DEPC-treated 0.1 M NaOH, DEPC-treated 0.05 M NaCl); Solution B (DEPC-treated 0.1 M NaCl);
  3. Proteinase inhibitor cocktail (Roche Diagnostics, Indianapolis, IN, USA)
  4. Halt™ Phosphatase Inhibitor (Thermo Fisher Scientific, Waltham, MA, USA)
  5. RNasin^Ⓡ^ Plus RNase Inhibitor (Promega, Madison, WI, USA)
  6. Protein binding buffer: 20 mM Hepes (pH 7.3), 50 mM KCL, 10% glycerol, 5 mM MgCl2. Add the following reagents before use: 1/1000 vol. of 1 M Dithiothreitol (DTT), Phosphatase inhibitor (100X), Proteinase inhibitor (100X), RNase Inhibitor (25~1000 U/mL, for RNA application).
  7. NuPAGE™ LDS Sample Buffer (4X) (Thermo Fisher Scientific, Waltham, MA, USA)
• Procedure

The procedure described below is an example of a single pull-down assay with one competition condition, calculate the volume of reagents needed according to the number of samples and the number of conditions to be processed. It is recommended to use 100 µL Dynabeads (10 mg/mL) per reaction with 500 µg~1000 µg total protein cell lysate (optimal concentration: 1 mg/ml).

Wash the Dynabeads magnetic beads prior to the pull-down assay according to “wash Dynabeads Magnetic beads” section. If use Dynabeads with RNA, please follow “Dynabeads magnetic beads for RNA manipulation” with additional treatment before immobilization steps.

I. Immobilize nucleic acids
  1. Resuspend beads in 2X B&W buffer to a final concentration of 5 µg/µL (twice original volume).
  2. Add an equal volume of the biotinylated-DNA/RNA in distilled water (this step is to dilute the NaCl concentration in the 2X B&W buffer from 2 M to 1 M for optimal binding).
  3. Incubate 15 min at room temperature using gentle rotation.
  4. Stand the mixture on magnetic stand for 2–3 min to collect the biotinylated DNA/RNA-conjugated beads.
  5. Resuspend the DNA/RNA-immobilized beads with 1X B&W buffer.
II. Pull-down of cytosolic DNA/RNA sensors/ binding proteins
  1. Preparation of cell lysate (cytoplasmic fractions): the following protocol is designed based on samples of approximately 8.8 × 10^6^ cells, which correspond to HeLa cells grown to confluence in a 100 mm tissue culture plate. Prepare PBS/Phosphatase inhibitor, Hypotonic buffer following the manufacturer's instructions (Nuclear Extract Kit from Active motif).
  1–1. Aspirate media out of dish. Wash with 5 mL ice-cold PBS/Phosphatase inhibitors. Remove the solution and add 3 mL ice-cold PBS/Phosphatase.
  1–2. Remove cells from dish by gently scraping with cell scrapers. Transfer cells to a pre-chilled 15 mL conical tube.
  1–3. Centrifuge cell suspension for 5 min at 500 rpm in a centrifuge pre-cooled at 4 °C.
  1–4. Gently resuspend cells in 500 µL 1X Hypotonic buffer by pipetting up and down several times. Transfer to a pre-chilled microcentrifuge tube. Incubate for 15 min on ice.
  1–5. Add 25 µL detergent (Nuclear Extract Kit from Active Motif) and vortex 10 s at highest setting.
  1–6. Centrifuge the suspension for 30 s at 14,000 X*g* in a microcentrifuge pre-cooled at 4 °C.
  1–7. Transfer supernatant (cytoplasmic fraction) into a pre-chilled microcentrifuge tube. Store the supernatant at −80 °C until ready to use.

2. Optional step (pre-clean): to remove some nonspecific binding to the beads, incubate cell lysate with unconjugated beads for 2 h at 4 °C with gentle rocking. Separate the beads with a magnet stand for 2–3 min, collect the pre-cleaned cell lysate for next step.
3. No competitor condition: Incubate 100 µL DNA/RNA-conjugated Dynabeads with the pre-cleaned cell lysate for 2~4 h at 4 °C with gentle rocking.
  With competitor condition: Add 10-fold excess amounts of none-biotinylated DNA/RNA in the pre-cleaned cell lysate and then incubate 100 µL DNA/RNA-immobilized Dynabeads with the cell lysate for 2~4 h at 4 °C with gentle rocking. For example, 200 pmole of oligonucleotide was immobilized with the Dynabeads, then we need 2 nmole oligonucleotide was added as a competitor.
4. Place the tubes on a magnet stand for 1 min and discard the supernatant.
5. Remove the tubes from a magnetic field and resuspend the beads in the protein binding buffer of 2~4 folds of initial volume of beads.
6. Repeated the washing steps for 3 times.
III. Elution with LDS sample buffer
1. Resuspend the beads with 20~40 µL 2X LDS sample buffer to each tube.
2. Boil the samples for 5 min.
3. After cooling down and gently vortex, put the tube on a magnet field for 1 min, transfer the supernatants to new tubes. The samples are ready for loading on sodium dodecyl sulfate-polyacrylamide gel electrophoresis (SDS-PAGE) for further analysis.

## Method validation

A pull-down assay using DNA/RNA-conjugated beads is widely used in various research fields, which is a direct and versatile tool to study DNA/RNA-protein interaction. In the method described here, we used Dynabeads^Ⓡ^ M-280 Streptavidin from Invitrogen. The strong binding affinity of the streptavidin-biotin interaction (Kd=10–15) is used in a vast number of applications [1,2]. We customized this method with a competition strategy, therefore improving the specificity and efficiency. The competitor of your choice can be same sequence as the conjugated-DNA/RNA, therefore conferring an information that which protein is specific for DNA/RNA binding protein through comparing the released proteins from the pull-down beads with or without competitor conditions; the competitor could also be any nucleic acids with totally different sequences from biotinylated-DNA/RNA, the result will suggest that if the competitor has competition binding with the biotinylated-DNA/RNA. The same competition strategy can also be applied to any other DNA/RNA-conjugated agarose [3,4], not limited to magnetic beads.

Using this customized DNA/RNA pull-down assay, our group previously reported that Ku70 is a novel cytosolic DNA sensor that induces type III rather than type I IFN [4]. In the report, a pull-down assay was performed using DNA or oligonucleotide-conjugated agarose beads (Thermo Fisher Scientific, Waltham, MA, USA). Cytosolic fractions from untreated HEK293 cells were incubated with the DNA beads conjugated with PCR-amplified, full-length pCR2.1. Proteins bound on the beads were separated on SDS-PAGE, followed by Coomassie blue staining. to determine DNA-specific binding proteins on the gel, 10-fold excess amounts of pCR2.1 (DNA competitor) were mixed with the cytosol fraction before incubation with the beads. The addition of the competitor DNA reproducibly led to the disappearance of protein bands at molecular mass 80 and 70 kDa, and later Zhang X. et al. confirmed that Ku70 is a novel cytosolic DNA binding protein.

Similarity, we have recently reported another new finding that siRNA enhances DNA-mediated IFN-λ1 induction through a crosstalk between DNA sensor IFI16 and RNA sensor RIG-I signaling pathway [5]. In this study, a series of pull-down assays using siRNA- or DNA-conjugated magnetic beads were utilized to identify siRNA and DNA sensor proteins in IFN-α–treated HeLa cells. Cytosolic fractions from IFN-α–treated HeLa cells were incubated with siRNA-conjugated magnetic beads with or without 10-fold excess amounts of Non-human Ctrl siRNA or plasmid DNA as competitors. The proteins bound to the beads with or without competitors were separated using SDS-PAGE and identified by MS analysis. The addition of the siRNA competitor led to the disappearance of the specific siRNA binding protein. The MS analysis identified RIG-I as the siRNA binding protein, which was further confirmed using western blot. When a 10-fold excess amounts of free siRNA was added as a competitor, the band corresponding to RIG-I completely disappeared. In contrast, when plasmid DNA was added as a competitor, the RIG-I protein band persisted, suggesting RIG-I specifically bound to siRNA. A similar set of studies were performed using DNA-conjugated beads. The result indicated that IFI16 was a DNA binding protein. The pull-down assays performed in this study with siRNA and DNA as competitors greatly help us illustrate a crosstalk of RIG-I and IFI16 signaling pathway.

More recently, in another study, biotinylated-DNA conjugated streptavidin beads were incubated with cytosolic fraction in the absence or presence of various siRNA competitors [6]. After incubation, proteins bounded to the beads were separated on SDS-PAGE and detected by western blot. In the pull-down assay, we added 10-fold excess amounts of Ctrl-siRNA, mm-siRNA (motif in the middle) or 3′m-siRNA (motif at 3′ terminus) as a competitor. The results indicated that DNA binding to DNA sensor IFI16 was not interrupted when Ctrl-siRNA or mm-siRNA was added as a competitor. However, we found that the band of IFI16 was diminished in the presence of the competitor, 3′m-siRNA, suggesting that 3′m-siRNA interrupts the binding of DNA to IFI16.

**Fig. 1:**
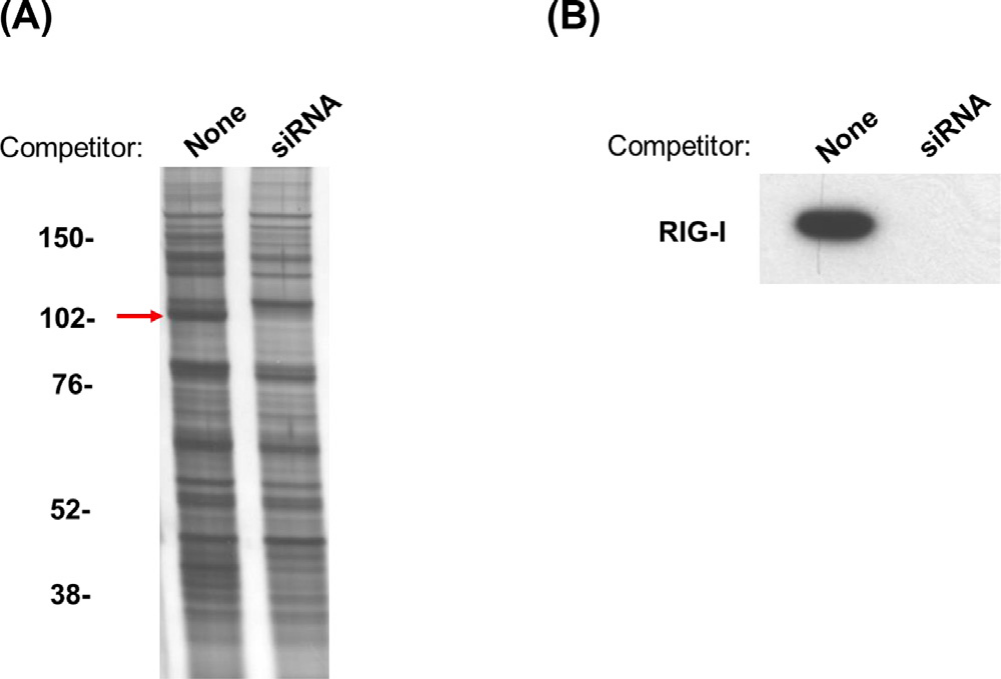
A biotinylated-siRNA-conjugated pull-down assay with the same sequence of double-stranded siRNA as a competitor. Cytosolic extracts of IFN-α–treated HeLa cells were incubated with siRNA-conjugated magnetic beads. Bound proteins on the beads were separated by SDS-PAGE gel, followed by **(A)** Silver staining and **(B)** Western blot using anti-RIG-I antibody.

To more clearly interpret the data from the described method in this report, we show here an example of the pull-down assay using siRNA-conjugated Dynabeads. Cytosolic fractions from IFNα-treated HeLa cells were incubated with the siRNA-conjugated beads. Proteins bound on the beads were separated on SDS-PAGE, followed by silver staining. to determine siRNA-specific binding proteins on the gel, 10-fold excess amounts of siRNA were mixed with the cytosol fraction before incubation with the beads. The addition of the competitor of siRNA reproducibly led to the disappearance of protein bands at molecular mass around 102 KDa, as shown in the Fig. 1(A). The MS analysis identified that the band is RIG-I as the siRNA binding protein, which was further confirmed using western blot. When a 10-fold excess amounts of siRNA was added as a competitor, the band corresponding to RIG-I completely disappeared, as shown in the Fig. 1(B).

In summary, our optimized protocol will help researchers more efficiently discover potential novel/ known DNA/RNA binding proteins or a new mechanism about the interaction between DNA/RNA and binding proteins.
